# Older Adults’ Continuance Intentions for Online Physical Exercise Classes

**DOI:** 10.3390/bs14050393

**Published:** 2024-05-08

**Authors:** Francisca Taveira, Belem Barbosa

**Affiliations:** School of Economics and Management, University of Porto, 4200-464 Porto, Portugal

**Keywords:** older adults, qualitative research, silver economy, online services

## Abstract

During the COVID-19 pandemic, lockdowns and social distancing measures drove the shift from in-person to online physical exercise classes, leading individuals to explore these digital alternatives. Guided by the Expectation-Confirmation Model, this article examines older adults’ intentions to continue using online physical exercise classes. Semi-structured interviews were conducted with 17 adults aged 65 and older who had participated in online physical exercise classes during the pandemic. Transcripts were subject to thematic analysis using the NVivo software program. The results indicate that older adults recognize the usefulness of online physical exercise classes because of their ability to enhance their health and well-being. Their initial expectations were surpassed, and they were generally satisfied with the experience. However, in-person classes remained preferred due to their enhanced benefits. They also felt that the adoption of online classes was involuntary; instead of an autonomous decision guided by their needs and preferences, this was a viable solution imposed by the lockdown. Therefore, their continuance intentions are limited to specific conditions, namely a new lockdown or other physical impediments. Still, considering the flexibility that online physical exercise classes offer, accommodating time and physical constraints, participants highlighted the advantages of a hybrid approach for those who may face challenges attending in-person classes. Based on the findings, this article proposes that ECM provides a relevant, yet insufficient, framework for explaining older adults’ continuance intentions for online physical exercise classes, suggesting the inclusion of additional explaining factors: perceived usefulness of non-technological alternatives, necessary conditions, and self-determination.

## 1. Introduction

The world population is aging at an unprecedented rate. According to the World Health Organization [1], by 2050, the number of people aged 65 and over is expected to outnumber children under five. In the case of the EU, this age segment is expected to almost double between 2020 and 2050. This prominent change in the demographic structure creates a higher demand for social and healthcare services, whose access and improved usage are critical to older people’s physical, social, and mental health [2]. The Organization for Economic Co-operation and Development (OECD) defines the elderly as people aged 65 and over, representing a growing segment of older consumers with new and distinct needs that must be addressed [3].

The Sustainability Development Goals (SDG) defined by the United Nations include the objective to “ensure healthy lives and promote well-being for all at all ages” [4]. In contrast to sedentary lifestyles, physical exercise is a crucial element of a healthy lifestyle that enhances the quality of life and physical and psychological well-being and fosters positive emotional and social interactions [5].

Recently, the COVID-19 pandemic imposed social isolation, leading to the widespread introduction of digital alternatives to face-to-face interactions. Several organizations, including the World Health Organization, recommended remote physical exercise practices through online platforms during isolation and social distancing. In fact, the pandemic has disrupted how individuals of all ages access digital services and interact with one another. Even older populations appreciated the value of technology, even though they historically used technology less frequently for everyday services and communication due to factors such as digital literacy and internet access. Overall, the increasing use of digital technology in daily activities and assisted living may facilitate a paradigm shift toward successful and healthy aging [6,7] and serve as an alternative to improving older individuals’ physical and mental health [8,9,10,11,12,13]. Therefore, it is essential to understand the impact of this digital shift on older adults’ health and wellness activities, including physical exercise.

As noted by Yap et al. [14], in the last few decades, researchers and practitioners have increasingly attempted to understand the factors that influence technology acceptance by the older population in e-commerce and e-health. However, the study of post-behavioral usage intentions regarding physical exercise in online contexts remains relatively unexplored. Recent contributions to the literature [9,10,15] have summarized the state of the art in online physical exercise classes for older individuals, highlighting a growing interest in the topic and stressing the urgent need for future research to address the remaining gaps. Dagenais et al. [9] and Gravesande et al. [10] conducted scoping reviews. Dagenais et al. [9] found that while existing literature explores the feasibility and the health-related impacts of online physical exercise classes, there is an urgent need to investigate further older adults’ experiences and perceptions associated with participation in such programs. Gravesande et al. [10], who specifically examined online mind–body programs for older adults, confirmed the feasibility and acceptance of this type of physical exercise class, noting that the research is often exploratory and focuses on technical barriers and facilitators, ease of use, and satisfaction of the participants.

Additionally, de Lacy-Vawdon et al. [15] synthesized the literature on factors that explain older adults’ engagement in group-based physical exercise classes. They identified critical elements such as the characteristics of the class, the instructor, the setting, social support, and perceived outcomes; however, they stressed the need for more high-quality studies, particularly regarding the methodological design. Moreover, the analysis of factors that explain continuance intentions is still lacking. Porter et al. [13] conducted a study with older adults who participated in online exercise classes during the pandemic and found that the majority were willing to continue participating in this type of class after that period. In line with these findings, a practical gap remains, as a clear understanding of the determinants of continuance intentions is necessary to guide practitioners and public stakeholders in their managerial decisions.

This article addresses these gaps by examining older adults’ intentions to continue using online physical exercise classes. As the focus of the article is the continuance intentions, it adopts the Expectation-Confirmation Model (ECM) as its theoretical underpinning.

This article has three main contributions. Firstly, it extends the literature on online physical exercise classes by exploring factors that explain continuance intentions among older adults. The study’s robust theoretical underpinning increases the relevance of the findings and provides a more substantial basis for future research. Secondly, this study also contributes to the scarce literature on technology acceptance by older individuals by exploring their experiences with online physical exercise classes. It further supports the adequacy of the ECM for explaining continuance intentions among older generations. It enables the identification of additional factors influencing technology continuance intentions: perceived usefulness of non-technological alternatives, necessary conditions, and voluntariness/self-determination. This finding is particularly interesting and can guide future research using ECM. Thirdly, this study provides relevant managerial contributions, as it offers evidence that can assist managers, policymakers, and other stakeholders interested in using technology to foster physical exercise among older generations to improve their well-being.

The remainder of the article is organized as follows. The next section provides a literature review on the ECM and explores the factors explaining intentions for post-behavioral usage. Section 3 presents the method adopted for this study, and Section 4 presents the results. Section 5 is devoted to the discussion of findings. Finally, Section 6 presents the conclusions, including implications, limitations of the study, and suggestions for future research.

## 2. Background

### 2.1. Expectation Confirmation Model (ECM)

The Expectation-Confirmation Model (ECM) was adopted to guide this research. It is a technology post-acceptance model and stands out among the theoretical frameworks on consumers’ post-adoption behaviors and intentions. As Mishra et al. [16] noted, the post-acceptance model is known in the academic literature by various names, including the expectation-confirmation model, information systems continuance theory, and information systems expectation-confirmation. For the purpose of this article, it will be referred to as ECM.

Bhattacherjee [17] developed the ECM as an adaptation of the Expectation-Confirmation Theory (ECT) initially proposed by Oliver [18]. In the Consumer Behavior literature, ECT is commonly applied to study consumer satisfaction and post-purchase behavior; it suggests that a consumer’s intention to repurchase a product or service is determined by their prior experience. As Oliver (1980) explained, whenever the benefit exceeds the consumer’s expectations, the resulting satisfaction leads to the continued intention to use it. By comparing their experience to their initial expectations, consumers will develop a favorable or negative impression of the product or service, affecting their satisfaction and, consequently, their willingness to repurchase the product or reuse the service.

Bhattacherjee [17] modified the ECT and proposed the Expectation Confirmation Model (ECM) as a post-acceptance model of information system continuance usage. In this context, continuance refers to individual users’ long-term use of technology beyond its initial adoption [19]. Bhattacherjee [17] suggested that users’ intention to continue using technology in the information systems domain resembled consumer repurchase decision-making behavior. From a long-term perspective, continued usage is essential for the technology to be truly accepted [17,20] and become a commercial success [16,21]. Compared to ECT, ECM places greater emphasis on post-adoption expectations than pre-adoption expectations. This is because a user’s expectations regarding technology use evolve with experience, and after assimilating these experiences, a user’s expectations may significantly differ from their initial assumptions [16,22].

Among the various perspectives examined in technology adoption research, perceived usefulness is the most consistent predictor of a user’s intentions [23] and serves as a proxy for post-adoption expectations. As a result, in the information systems’ setting, post-use perceived usefulness (PU) replaced the ECT expectation concept, and repurchase intention was also replaced by continued usage intention. Furthermore, and more crucially, the ECM defined confirmation as “the congruence between expectation and actual performance” [17] and eliminated the performance construct of ECT since the ECM considers that confirmation already explains the effect of perceived performance [17].

Hence, ECM asserts that an individual’s continuance intention in using an information system depends on three variables: satisfaction, the degree of confirming users’ expectations, and the users’ post-usage beliefs regarding the benefits of the information system in the form of perceived usefulness [22].

The ECM is widely used in a variety of areas, including e-learning [24], e-commerce [21,25,26,27,28,29], m-commerce [30,31], s-commerce [32], shared transportation services [33,34,35], social media [36,37], e-banking services [38,39,40,41], wearable technology [42], and e-health systems [43,44,45,46]. Moreover, ECM has been proven beneficial in explaining topics related to fitness, namely fitness and health apps [46], and also in studies on older consumers, namely the adoption of e-banking [39] and social media [37].

### 2.2. Factors Explaining Post-Behavioral Usage Intentions

#### 2.2.1. Post-Acceptance Perceived Usefulness

The literature frequently emphasizes perceived outcomes as critical factors influencing older adults’ participation in online exercise classes [9,10,15]. ECM suggests that perceived usefulness is essential to understanding satisfaction and continuance intentions. Post-acceptance perceived usefulness (PU) reflects post-usage beliefs and refers to the users’ perceptions regarding the benefits of using a system, service, or technological device [17]. Thus, it represents the expectation outlined as a belief about the importance of using certain technologies and serves as a point of comparison [22]. Users’ beliefs and perceptions about technology shift throughout time and continue to change as they become more familiar with the technology [22]. It has been studied that the influence of PU on satisfaction decreases for long-term users since they have already gained a reasonable level of information about the systems. PU has been shown to improve satisfaction levels, particularly among early adopters and short-term users [16,47].

In a study that applied ECM, Chiu et al. [46] confirmed that post-acceptance perceived usefulness is a crucial determinant of older adults’ continuance intention to use fitness and health apps, namely because it has a positive impact on users’ satisfaction with the technology. It is worth noting that their sample included individuals of all ages and primarily involved users aged 26 to 35, leaving the perspectives of older users unexplored. However, studies that evaluate the feasibility of online physical exercise classes for older adults tend to highlight their usefulness, namely in terms of the physical well-being of the participants [10,13], demonstrating the importance of this variable in understanding continuance intentions.

#### 2.2.2. Confirmation

Confirmation is the degree to which an individual’s original expectation about a system’s performance is confirmed after having an experience with the system [18]. These perceptions are shaped by comparing users’ initial expectations and the post-usage benefits to create the confirmation/disconfirmation status regarding the reuse of the system in users’ minds [17]. In the Expectation-Confirmation Model, confirmation describes an individual’s affective state as a consequence of a cognitive assessment of the potential discrepancy between initial expectation and experienced performance [16,17]. To become successful, information systems should be capable of satisfying users by meeting their expectations through expected usefulness and benefits [16].

To the best of the authors’ knowledge, the literature has not addressed confirmation in the context of online physical exercise classes, particularly regarding older adults. A relevant contribution was offered by Leung and Chen [44], who conducted a study on e-health/m-health adoption and lifestyle improvement in the general population of Hong Kong. They found that users’ degree of confirmation was positively associated with satisfaction with health-related information platforms/apps, since once the initial expectation is met or exceeded, consumers are more likely to foster a positive feeling toward or be satisfied with the new technology and thus continue to use it [44]. A similar result was observed by Chiu et al. [46] in their study on fitness and health apps conducted in China, with the majority of participants being between 26 and 35 years old. They found that it positively affected user satisfaction, subsequently increasing the intention to continue using the apps. Hence, these studies empirically supported ECM assumptions on health-related topics. The gap remains regarding older adults, particularly online physical exercise classes.

#### 2.2.3. Satisfaction

Lastly, satisfaction generally represents an individual’s level of approval resulting from involvement in an activity, event, or matter. Bhattacherjee [17] defined this construct as “a psychological or affective state related to and resulting from a cognitive appraisal of the expectation-performance discrepancy (confirmation)” (p. 354). Individuals experience pleasure when information systems meet or surpass their expectations in terms of functionality, performance, and benefits [16]. This post-acceptance satisfaction is grounded in users’ first-hand experience and is, therefore, more realistic, unbiased, and less susceptible to change.

As Mishra et al. [16] noted, the literature generally supports the positive relationship between satisfaction with various information systems (e.g., social media, online apps) and usage continuance intentions. As mentioned above, this positive relationship was confirmed among users of e-health/m-health apps [44] and e-fitness apps [46] within general populations in Hong Kong and China, respectively. Furthermore, Cronin et al. [48] conducted a study with physiotherapy patients aged between 18 and 79 who participated in an online exercise program during the pandemic and found high levels of satisfaction that could be associated with positive intentions to use hybrid and online physiotherapy sessions. Gravesande et al. [10] and Dagenais et al. [9] stress that, generally, the literature shows that online physical exercise classes provide high satisfaction levels among older adults. Hence, it is essential to, in line with what was postulated by ECM, further explore the role of satisfaction with online physical exercise classes to explain continuance intentions.

## 3. Method

This study employed a qualitative exploratory approach, which aims to better understand the researched topic by exploring the experiences and perspectives of individuals [49]. This qualitative study relies on information gathered from semi-structured interviews with older adults (aged over 65, of both genders) who participated in online physical exercise classes during the COVID-19 pandemic. The interviews were conducted in May 2023.

### 3.1. Materials

The interview script was then divided into three main topics: (i) contextualization of the physical exercise experience before the pandemic, (ii) transitioning to online physical exercise classes during the pandemic, and (iii) exploring prospects for online physical exercise classes. An outline of the main questions is presented in Appendix A. Before the study, a pilot interview was conducted to assess the clarity of the questions and the consistency of the sequence. During each interview, the interviewer began with general questions and asked additional ones when necessary to elicit further details.

The researchers obtained informed consent from interested participants to ensure that participants fully understood the research objectives, procedures, risks, and benefits. The study was conducted in accordance with the “Declaration of Helsinki”, following the ethical principles recommended for social research as outlined by Bryman [49], particularly informed consent, confidentiality, anonymity, and voluntariness. Participation in this study was entirely voluntary. Participants were informed that they had the right to decline participation and could withdraw at any time during the study. Data confidentiality and anonymity were guaranteed throughout all phases of the study.

### 3.2. Participants

The participants were selected through purposive sampling. As explained by Bryman [49], in purposive sampling, participants are strategically selected based on their experience and relevance to the research rather than through random sampling. Thus, the sample is of a convenience rather than a probabilistic nature. Participants were recruited based on the researchers’ professional and personal networks, namely through a community exercise program offered by the University of Porto for individuals aged 65 or older. This program, which provided online physical exercise classes during the pandemic, qualified its participants for this study. A snowball sampling technique was also employed, wherein participants were asked to suggest other potential interviewees. The final sample (Table 1) comprised 17 volunteers aged 65 and older who participated in online physical exercise classes during the COVID-19 pandemic, representing diverse sociodemographic and urban characteristics. The sample size was deemed suitable for a qualitative study [49], as it allowed data saturation to be achieved, i.e., the final interviews did not provide any new information or themes, making additional data collection redundant. Since the research objectives and the study population were very specific, data saturation tends to require a relatively small sample size [49]. It should be noted that sample sizes in qualitative studies vary significantly. Unlike quantitative research, which relies on statistical power calculations to determine sample size, qualitative studies focus on collecting rich, detailed data to understand the subject comprehensively. Qualitative studies do not aim to generalize findings [49]. In narrow-scope studies with specific populations, sample sizes can be under two digits and frequently less than twenty as long as data saturation is achieved [49]. In this study, data saturation was achieved in the fourteenth interview.

### 3.3. Data Analysis Procedures

The interviews were recorded using an encrypted voice recorder, and the transcripts were systematically analyzed through thematic content analysis [49,50,51]. Overall, content analysis aims to uncover themes, patterns, and meanings from qualitative data. The identification of patterns and data codification were performed with the software tool NVivo (Version 12, QSR International, Southport, UK). The study employed a priori and posteriori categories in content analysis, starting with a theoretical framework and progressively refining our outcomes to align with the research objectives [50]. According to Hsieh and Shannon [52], inductive data-driven content analysis is particularly useful for underexplored topics with limited existing literature, as with this article. As recommended by the literature [49,50,53], coding was performed after data preparation procedures (e.g., verbatim transcription and anonymizing the data). The data were organized into main categories and subcategories, associating transcript passages with codes and identifying recurring themes and patterns. The analysis was followed by validation and reporting. Although software tools like NVivo can perform basic statistical analyses based on word counts (e.g., word frequency, coding frequencies, charts, and graphs), this analysis prioritizes interpreting meanings. Therefore, the results do not include quantitative approaches to the qualitative data.

## 4. Results

As indicated in the preceding section, every participant in this study had engaged in online physical exercise classes during the COVID-19 pandemic lockdown. This period was marked by substantial challenges due to the widespread transmission of the coronavirus. Social distancing measures led to the closure of public sports facilities, including gyms. Several providers offered online exercise classes during that period to keep their customers engaged in physical activity, which all participants in this study chose to experience.

However, participants emphasized that adopting online physical exercise classes was not perceived as totally voluntary as, during the lockdown, this was the only alternative left for maintaining physical exercise routines. Overall, they perceived this alternative as an opportunity to exercise, socialize, and use their time.

“We couldn’t leave the house. At that time, we were obliged to be in front of the computer…”

P9

“And I enrolled [in online physical exercise classes] with pleasure, to socialize and keep up with the exercise, to maintain the discipline.”

P5

“We saw our colleagues, we did something. It was not as good as being there, but at least we did something so that we wouldn’t be sedentary for too long.”

P3

These findings align with Newbold et al. [54], who argue that the emergence of online physical exercise programs and recorded classes facilitated physical exercise routines despite lockdowns, social isolation, and gym closures.

In the following sections, we explore the participants’ experience with online physical exercise classes and their continuance intentions.

### 4.1. Participants’ Experiences with Online Physical Exercise Classes

Most participants had low or no initial expectations for online physical exercise classes due to their unexpected adoption. However, their overall experience was generally positive, as explained by the following transcripts.

“It exceeded expectations. Honestly, I had no idea what an online class was like; it was a whole new world. (…) Given the circumstances, nothing was missing, with limitations, but nothing was missing.”

P3

“I never thought it would work, and I think it worked very well. I know that my colleagues were also very happy to have been able to do it.”

P16

Similarly, the emotions conveyed during the interviews were very positive, as most participants expressed contentment and appreciation regarding the experience.

“The gym offered this solution, which was great. It was different, but it was interesting.”

P8

“It was one of the best things [during the pandemic]. I loved it.”

P3

“It was great. Everything went well, and we were very well guided by the fitness instructor. I think we all collaborated very well; there were no flaws, nothing.”

P12

“I liked it, of course. If I hadn’t liked it, I would have quit.”

P2

According to the literature [17], and as participants pointed out, satisfaction fosters a positive relationship between the user and the technology, encouraging repeated usage and sustained adoption. However, if participants, such as P6, do not perceive benefits from the online service and have initial negative expectations, they may confirm those low expectations, resulting in an overall negative evaluation of the online classes.

“I never thought I would like [online physical exercise] classes, and I really didn’t. I felt like I didn’t gain anything that would make me want to attend the class. We were just at home, moving from one place to another to do the exercises. But it tired me out, and I would leave.”

P6

In accordance with Mishra et al. [16], positive experiences and satisfaction encourage users to use technology more, whereas negative experiences may lead to technology discontinuation or avoidance.

### 4.2. Perceived Usefulness

The primary motivation for the participants’ involvement in physical exercise classes was their health and well-being. Their experiences with online classes during the COVID-19 pandemic proved particularly useful, as these virtual alternatives allowed them to maintain physical activity and contribute to their overall well-being. Several participants, such as P3, expressed appreciation for this opportunity amidst the difficult times of lockdown and social isolation.

“It was essential to keep us from being idle for too long. It was like giving a toy to a child who had nothing.”

P3

Besides the physical and emotional benefits of continuing their physical exercise activities, one factor that stood out as an explanation for satisfaction with the experience of online physical exercise classes was their perceived usefulness, particularly in the context of lockdown and social distancing.

“It was very useful because we were confined at home for a long time, and it seemed likely to continue. So, it was useful in the sense that it kept us engaged in an activity similar to what we were accustomed to doing.”

P17

“As we had to stay at home, which was mandatory, [having online physical exercise classes] was a privilege; we could stay home and keep exercising.”

P14

Moreover, participants pointed out the general advantages of online classes that demonstrate their usefulness beyond lockdown periods, particularly their convenience. One of the participants suggested that online classes could be a solution to avoid absenteeism, especially in bad weather conditions.

“The advantages include the convenience of being in our own home, or any preferred location, where we feel comfortable and accustomed, eliminating the need for commuting. We don’t have to travel, whether by public transport, private vehicles, or on foot.”

P10

“Another advantage of online was that it could rain heavily, and we wouldn’t miss a class. Because when it rains a lot, we usually skip class.”

P9

As highlighted by our participants, mobility challenges and limited access to reliable transportation often prevent older adults from attending in-person classes. By eliminating the need to travel, online fitness programs make exercise more accessible to those who participate. These findings corroborate the points made by da Silva et al. [55], who stressed that the convenience of exercising at home, the ability to avoid adverse weather conditions, and not facing transportation issues are significant advantages. Porter et al. [13] further suggest that convenience may lead older adults to favor the flexibility and accessibility of online options, even in the post-pandemic period. The following section explores, in detail, continuance intentions.

### 4.3. Continuance Intentions

The possibility of participating in online physical exercise classes in the future was presented as promising, yet as a last resort. This idea was shared even by participants who enjoyed online physical exercise classes, as was the case with P1.

“If there were no other option, I would recommend it. But if there were [in-person classes], I would forget about the online. Even though I did it and liked it, it doesn’t mean I would prefer it…”

P1

Generally, participants felt that the benefits of in-person classes outweighed those of online ones. Despite the specific advantages of online classes, they prefer in-person sessions and would consider online options only if in-person participation becomes impossible for some reason.

“Anything can happen. There are so many online classes now. It’s a resource; it’s never wasted time, but it’s not as beneficial as in-person classes.”

P11

“If I have to do it, I will. But not voluntarily…”

P15

“[I would consider it] only when I really can’t come [to in-person classes] anymore because as long as I can, I prefer [in-person]. Because we have other things. We have machines and more diversified exercises. I think it’s more useful here than at home.”

P4

Specific scenarios were identified in which this online format might be beneficial. Most participants recognized online physical exercise classes as useful and valuable. They suggested that these classes are particularly suitable for older adults with limited mobility, those living in distant locations, individuals who prefer to avoid commuting, or those caring for someone at home. The participants in this study suggested that individuals with certain health conditions or physical limitations might feel more comfortable and confident participating in online fitness programs. Yet, they generally did not favor this solution for themselves—at least for the time being, as they have the physical and material conditions to attend in-person classes.

“I don’t live nearby. I use public transport, which can still be expensive because I have to buy a monthly pass and all that. But it’s an investment I’m willing to make. Coming here and taking classes gives me pleasure. Online is out of the question.”

P6

According to the literature (e.g., [56]), when older adults perceive the usefulness of online physical exercise classes, they are more likely to recommend them to others, such as friends and family, thereby contributing to the favorable reputation of these programs. This was evident in most of the interviews. Participants who enjoyed the experience and acknowledged the usefulness of online physical exercise classes indicated they would recommend them to others, especially those with any impediment to attending in-person classes.

“I really prefer in-person classes. For those who can’t make it, it’s great. If a person has more difficulty, transportation issues… there are multiple situations, living far away… then, yes, I think it’s better through the computer.”

P8

“I would highly recommend it to those who can’t come. People who don’t have transportation or can’t dislocate for any other reason, perhaps they may have sick family members… I would strongly advise them to take these online classes.”

P12

Ultimately, while online physical exercise classes offer numerous advantages, it is important to emphasize that they are most effective when used with in-person classes or activities. A hybrid approach combining both online and in-person options can allow older adults to enjoy the benefits of flexibility while also benefiting from the social interaction that in-person classes provide [13].

## 5. Discussion

**Figure 1:**
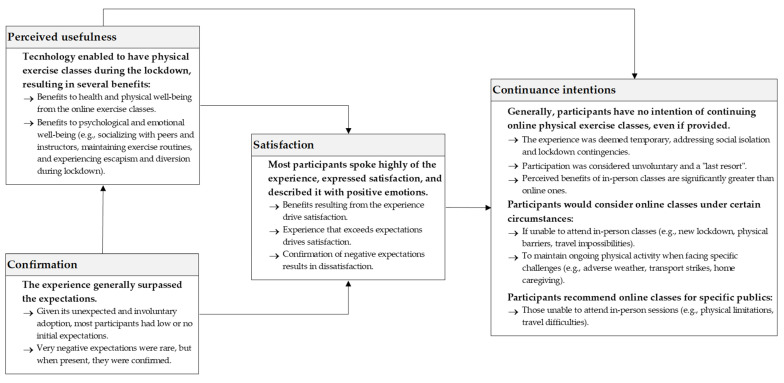
Summary of findings.

Figure 1 summarizes the findings of this study. The scheme is adapted from Bhattacherjee [17] and his post-acceptance model of usage continuance to organize our findings.

In line with the theoretical underpinnings [17,18], this study confirmed that perceived usefulness and confirmation determine satisfaction, providing additional evidence on the adequacy of ECM to understand continuance intentions. Most participants shared positive experiences with online physical exercise classes, attesting to their satisfaction. This fact was primarily attributed to the usefulness of these classes in overcoming limitations imposed during the pandemic. Furthermore, satisfaction correlated with the confirmation of initial expectations, even among individuals who had very negative initial expectations and confirmed them during the experience. Hence, the basic relationships proposed by ECM [17] between perceived usefulness, confirmation, and satisfaction provide a relevant lens to analyze the participants’ experience with online physical exercise classes.

However, this study did not evidence the expected positive association between satisfaction and continuance intentions. Participants generally showed no intention of continuing with online physical exercise classes, even if they were available. The explanation is twofold. Firstly, participants did not consider their adoption of online physical exercise classes voluntary. To some extent, they felt forced to adopt this modality. This is an interesting result since anyone who decided to engage in online exercise could decline. All participants in this study had experience with this class type, whereas not all of their peers chose to participate. The feeling of involuntariness stems from the fact that the lockdown circumstances determined the solution. Even those who enjoyed and praised the experience emphasized that it was a “last resort” solution. Secondly, despite recognizing this solution’s usefulness and its general benefits, participants also stressed their preference for in-person classes, which offer additional benefits and advantages.

Yet, participants would consider participating in online physical exercise classes again under certain circumstances, not just in the event of new lockdowns. They indicated that online classes could be a reasonable alternative for times when they are unable, for various reasons, to go to the gym (e.g., adverse weather, transport strikes, or physical limitations). Accordingly, they suggested that online classes are a good option for other individuals facing these limitations.

As such, this study contributes to the technology continuance intention literature, as it found three complements of the ECM model to explain continuance intentions. Firstly, contextual factors, particularly the lockdown and physical impediments, were highlighted as a condition for the continuance intentions regarding online physical exercise classes. Participants mentioned that they would continue if a new lockdown was imposed or if they could not participate in person. The arguments provided by the participants were coherent with the necessary condition logic proposed by Dul [57], which analyzes the importance of specific conditions essential for achieving a particular outcome. However, it is not sufficient by itself to trigger it. Hence, the physical impediment to attending in-person classes was described as a necessary yet insufficient condition for online class continuation attendance.

Secondly, while the perceived usefulness of online physical exercise classes is evident, participants’ continuance intentions are shaped by the comparison with available alternatives, particularly in-person classes. The participants indicated that, although online classes were valuable, the face-to-face format not only provides additional benefits (e.g., access to equipment, diverse modalities, personal support from the fitness instructor) but also amplifies similar benefits to a greater extent (e.g., socialization, health, and wellness benefits). Therefore, the usefulness of in-person classes is perceived as significantly superior. While ECM considers the perceived usefulness of technology, it overlooks its comparison with non-technological alternatives.

Thirdly, an unavoidable factor in explaining the participants’ behavior was that the choice for online classes was not voluntary, as they repeatedly stated—this was the “last-resort” alternative they had during the lockdown. This result can be interpreted through the Self-Determination Theory (SDT) lens. As explained by Ryan and Deci [58], human behavior can be classified according to diverse levels of autonomy, ranging from external regulation, according to which behaviors are controlled by extrinsic factors, to integrated regulation, according to which the decisions are congruent to individual goals and needs. As stressed by Webb et al. [59], SDT suggests that any autonomously motivated behavior is more likely to endure over time and consequently be associated with higher continuance intentions.

**Figure 2:**
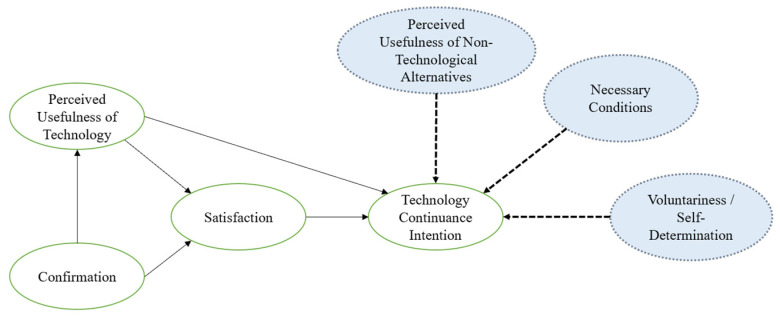
Proposed adaptation of the ECM model based on the findings. Note: The circles in green represent the original variables of the ECM model. Circles in blue are contributions of this study.

Consequently, this study suggests that to explain the continuance intentions to attend online physical exercise classes, the ECM model should be extended by incorporating three additional factors: perceived usefulness of non-technological alternatives, necessary conditions, and voluntariness/self-determination (Figure 2).

## 6. Conclusions

During the COVID-19 pandemic, the shift from in-person to online physical exercise classes was driven by lockdowns and social distancing measures, leading individuals to explore these digital alternatives. Our research indicates that older adults recognize the usefulness of online physical exercise classes, namely because of their ability to enhance their health and well-being; their expectations were surpassed; and they were generally satisfied with the experience. However, in-person classes remained preferred due to their enhanced benefits. They also felt that adopting online classes was involuntary; instead of an autonomous decision guided by their needs and preferences, this was a viable solution imposed by the lockdown. Consequently, their continuance intentions are limited to specific conditions, namely a new lockdown or other physical impediments. Still, considering the flexibility that online physical exercise classes offer, accommodating time and physical constraints, participants highlighted the advantages of a hybrid approach, accommodating those who face challenges in attending in-person classes.

Hence, the innovations emerging during this period have expanded opportunities for staying active and are expected to remain valuable as circumstances evolve. As such, online physical exercise classes can help maintain an active lifestyle by facilitating and democratizing access to physical exercise classes, contributing to older adults’ sustainable health and well-being.

### 6.1. Theoretical Contributions

Yap et al. [14] have highlighted the lack of attention given to the specific service of online physical exercise classes and the factors influencing older adults’ participation and engagement decisions. Additionally, recent reviews of online physical exercise classes for older adults [9,10,15] have uncovered a scarcity of studies, particularly those exploring participants’ experiences. Although there is some evidence that older adults who have participated in online physical exercise classes reported high satisfaction levels and some willingness to continue using this type of class [13], to the best of the authors’ knowledge, a study focusing on the determinants of continuance intentions is still lacking. This study provides valuable contributions to this field of research. It offers rich empirical data, contributing to the literature on online physical exercise classes. It enables a deeper understanding of seniors’ experiences, including factors that influence their continuance decisions in this context. It also demonstrates the adequacy of the ECM in explaining consumer satisfaction. Furthermore, it highlights the limitations of the model in explaining continuance intentions in the case of technological adoption, which individuals feel are involuntary and a last resource. After the experience, they consider the non-technological alternative to be more advantageous. Also, the study identified the existence of necessary conditions to explain continuance intentions. As a result, this study makes a relevant contribution to the technology continuance intention literature by suggesting the inclusion of three additional factors to explain continuance intentions: perceived usefulness of non-technological alternatives, necessary conditions, and voluntariness/self-determination. In this regard, it stresses the possible complementarities between ECM, SDT, and necessary condition logic, contributing to future ECM model developments.

### 6.2. Practical Implications

Participants who have experienced both in-person and online physical exercise classes tend to prefer in-person interactions. However, the study highlights the timeliness of offering online classes for certain circumstances, leading this study to recommend the continuation of hybrid class models, which combine both formats. A hybrid model provides flexibility, expanding the potential audience by accommodating those who prefer the convenience of online classes while catering to those who value personal interactions. This approach also addresses mobility, location, and scheduling challenges some individuals face, thus enhancing their overall experience. Ultimately, offering online classes complements in-person offerings, increasing consumer loyalty and innovation and aligning with market demands. It also presents fitness centers with opportunities to enhance their business image.

This study also allows for important conclusions to be drawn for policymakers and those responsible for public policies, particularly in the direction of increasing physical activity and health, such as financial support for digital inclusion, technology training for seniors, the promotion of accessible online exercise programs, and the creation of participation incentives.

### 6.3. Limitations and Future Research Directions

Some limitations of this study must be acknowledged. First, this study is exploratory and includes a limited number of participants recruited using a convenience sampling technique. However, the sample size was considered adequate for this study, as participants have diversified profiles, the data captures diverse experiences and perspectives, and data saturation was achieved. Given its qualitative and exploratory nature, this study’s findings are linked to the participants’ context, views, and experiences and are not intended to be generalizable to a broader population. The extrapolation of the findings requires further validation, namely by replicating the study with distinct samples.

Future research may also consider different methodological approaches. This could involve using both qualitative and quantitative methods to provide comprehensive and detailed data collection methods and establish causal relationships. Another area for additional research could be examining older adults’ actual ongoing usage behavior. Longitudinal studies that track participants’ experiences and participation over time may assist researchers in determining the long-term impact.

Finally, this study proposes the inclusion of new factors to explain continuance intentions. The quantitative test of the underlying hypotheses is beyond the scope of this study and should be addressed by future research. This could be conducted in the context of online physical exercise classes and other online services that gained popularity during the pandemic. Comparisons between different age groups could also provide valuable contributions to the literature and generate relevant managerial and societal implications.

## Figures and Tables

**Table 1:** Participants.

Code	Age	Gender	Education Level	Type of Online Class	Interview Duration	Number of Words
P1	73	Male	Primary	Aerobics	18:04	1415
P2	89	Male	Primary	Aerobics	12:49	1021
P2	75	Female	Primary	Aerobics	16:22	1083
P4	74	Male	Primary	Aerobics	14:10	1040
P5	72	Male	Secondary	Aerobics	17:09	1346
P6	83	Female	Primary	Aerobics	19:46	1357
P7	81	Female	Primary	Aerobics	16:36	1154
P8	77	Female	Secondary	Pilates	18:26	1494
P9	80	Male	Secondary	Pilates	24:33	1886
P10	72	Male	Secondary	Aerobics	27:33	2013
P11	82	Female	Higher education	Pilates	20:02	1258
P12	77	Female	Primary	Pilates	19:59	1256
P13	79	Female	Higher education	Aerobics	17:39	1052
P14	71	Female	Primary	Pilates; Aerobics	20:22	1368
P15	77	Male	Secondary	Pilates	23:19	1804
P16	74	Female	Higher education	Pilates	13:21	1002
P17	85	Male	Higher education	Pilates	22:53	1631
Total					05:23:03	23,180

## Data Availability

Data are contained within the article.

## Appendix A. Interview Outline

1. Did your gym provide online exercise classes during the lockdown? Can you tell me more about this?
2. Overall, how would you describe your experience with online exercise classes?
  2.1 What were the main challenges you faced with online exercise classes?
  2.2 What do you consider the main advantages of online fitness classes?
  2.3 What are the main disadvantages of online exercise classes?
  2.4 Were your expectations met?
  2.5 What benefits did you gain from the online fitness classes?
3. What is your current opinion on online fitness classes?
  3.1 Would you recommend online fitness classes, and if so, to whom or in what situations?
  3.2 Will you likely participate in online fitness classes again, and if so, under what circumstances?
  3.3 How do you think online fitness classes could be improved?
